# Offsets in tide-gauge reference levels detected by satellite altimetry: ten case studies

**DOI:** 10.1007/s00190-023-01800-7

**Published:** 2023-12-03

**Authors:** R. D. Ray, M. J. Widlansky, A. S. Genz, P. R. Thompson

**Affiliations:** 1 NASA Goddard Space Flight Center, Greenbelt, MD USA; 2 Cooperative Institute for Marine and Atmospheric Research, School of Ocean and Earth Science and Technology, University of Hawai‘i at Mānoa, Honolulu, HI USA; 3 NOAA’s National Centers for Environmental Information, Honolulu, HI USA; 4 Department of Oceanography, School of Ocean and Earth Science and Technology, University of Hawai‘i at Mānoa, Honolulu, HI USA

**Keywords:** Tide-gauge datum, Sea level, Satellite altimetry, Vertical land motion

## Abstract

Comparing measurements of absolute sea level by satellite altimetry and relative sea level by a tide gauge can reveal errors in either measurement system. Combining the measurements can determine vertical land motion (VLM) at the tide gauge. We here discuss ten case studies in which a tide gauge has likely experienced a small ($\le 10$ cm), discontinuous offset in the vertical, suggesting inadvertent loss of reference-level stability. Proper interpretation of offsets is helped if independent VLM measurements from nearby geodetic stations are available. In two cases, earthquake-induced VLM cannot be ruled out, although it appears unlikely. Offsets as small as 1–2 cm can be detected when both altimeter and tide gauge successfully observe the same ocean signal. This is most likely to occur for tide gauges located on small, open-ocean islands. Tide gauges near large land masses are typically more challenging owing to inadequacies of satellite altimetry near land and to differences between coastal and open-ocean sea levels. The case studies highlight the utility of satellite altimetry for tide-gauge quality control.

**Supplementary Information:**

The online version contains supplementary material available at 10.1007/s00190-023-01800-7.

## Introduction

The global network of tide gauges has long been used to assess and monitor the accuracy of satellite altimetry (e.g., Cheney et al. 1994; Mitchum 1998; Ablain et al. 2017). Several times over the past three decades the tide-gauge network has helped the altimeter community identify instrument problems, such as a drifting water-vapor radiometer, or even data-processing problems (Nerem 1997; Fu and Haines 2013). The tide-gauge network is an essential tool for monitoring stability and establishing uncertainty in altimeter observations.

The reverse is also true: satellite altimetry can help identify problems at tide gauges. In this work we investigate the use of altimetry for monitoring the long-term vertical leveling control of individual tide gauges. We discuss ten case studies in which problems in tide-gauge reference levels, or station datums, have probably occurred. In fact, satellite altimetry is already routinely employed for validation purposes in some sea-level data centers, and it is explicitly called for in the quality-control manuals of the Intergovernmental Oceanographic Commission (UNESCO/IOC 2020). The method is routinely used at the University of Hawai‘i Sea Level Center (UHSLC). As an example, in the Joint Archive for Sea Level (JASL) Research Quality (RQ) dataset, housed at UHSLC, the documentation for the Rodrigues, Mauritius, tide-gauge record notes a possible reference-level shift that was discovered by comparing with satellite altimetry; we revisit this case below.

Reference-level offsets at tide gauges most commonly occur because of reinstallation of sensors. The need for reinstallation can arise for many reasons, including destruction of a station by storms, the need to move a station for harbor construction, replacement of a malfunctioning sensor, or replacement with an upgraded sensor. When tide-gauge sensors are reinstalled, especially in high-quality networks (e.g., those associated with the JASL and the Global Sea Level Observing System), every effort is made to reconnect the observations to the existing local elevation benchmark network. Yet the leveling process introduces uncertainty and human errors are possible, so detection of reference-level errors is an ongoing concern. The employment of altimetry in this cause can be invaluable.

In addition to their mutual use for quality control, the combination of tide gauges and satellite altimetry forms a powerful geodetic tool for determining vertical land motion (VLM). Because altimetry measures absolute sea level in a geocentric reference frame, while tide gauges measure relative sea level with respect to local benchmarks on land, their difference (hereafter referred to as “Alt–TG”) yields a time series of vertical position of the gauge in the satellite reference frame (e.g., Cazenave et al. 1999; Nerem and Mitchum 2002; Fenoglio-Marc et al. 2004; Ray et al. 2010; Ostanciaux et al. 2012; Wöppelmann and Marcos 2016; Pfeffer and Allemand 2016; Kleinherenbrink et al. 2018; Oelsmann et al. 2021). This approach to determining VLM is capable of capturing both abrupt co-seismic vertical offsets and gradual post-seismic deformation (e.g., Han et al. 2019). The method works only when both the altimeter and tide gauge are observing the same ocean signal, and this is its main limitation, which we discuss further below.

In this work, our analysis of tide-gauge datum problems is integrally tied to our estimation of VLM via Alt–TG differences. We examine VLM in terms of both its internal consistency within a time series and its external consistency with independent geodetic VLM measurements.

When abrupt jumps in VLM arise from earthquakes, volcanic processes, or other rapid ground displacements, the resulting abrupt changes in relative sea level are obviously not tide-gauge errors. Such jumps must be distinguished from the reference-level offsets that are our concern here. Altimetry alone cannot solve that issue, and independent information is needed. That will be a recurring theme in the discussions below.

Here we explore ten previously unresolved cases (similar to Rodrigues, Mauritius) of abrupt and likely non-geophysical offsets in the UHSLC RQ dataset as suggested by Alt–TG analysis, thereby emphasizing the importance of altimetry for quality control of tide-gauge data. To be clear, most of the tide-gauge records in the UHSLC archives do not show detectable datum errors. Furthermore, it is also true that the errors we are studying, often only a few cm, can be difficult to detect, especially in locations where sea-level variability is large.

When a non-geophysical offset in tide-gauge data is identified via the Alt–TG approach, one faces a decision about how to resolve it, which leads to an important “philosophical” point about the independence of measurement systems. When validating altimeter measurements using tide gauges (e.g., Mitchum 1998), the goal is to identify potential problems as revealed by Alt–TG differences and then to investigate the satellite systems in detail to search for the cause(s) and correct as needed. The tide-gauge data remain independent. That is the validation approach advocated by Beckley et al. (2017) and by others. We admit the approach is not universally followed, since some investigators have used tide-gauge data to derive altimeter “corrections”—and in fact the DUACS altimetry used extensively in the analysis presented here does this for one period (see discussion below). The philosophy of system independence, if accepted, applies equally well to the reverse problem: when altimetry suggests a problem with a tide gauge, the best approach is to attempt to locate and correct the cause, using whatever documentation exists for deployments, levelings, adjustments, etc., and whatever equipment inspections are appropriate.

The ability to maintain system independence depends on detailed understanding of—and physical access to—the tide gauges being investigated. That is why seven of the ten case studies herein involve tide gauges that are maintained and quality-controlled by the UHSLC. For these stations, we have ready access to field documentation, including elevation surveys performed during maintenance visits, which can potentially illuminate the ultimate cause(s) of non-geophysical vertical offsets. In one of these cases, our analysis led to discovery of a definitive instrumental problem which needed to be—and was—corrected. The remaining three cases involving non-UHSLC tide gauges are included because the Alt–TG method allowed us to identify problems that we subsequently discovered had already been corrected by data originators (in two cases based on earlier correspondence with UHSLC) but were not yet synchronized with the JASL RQ dataset. Otherwise, we do not address problems in non-UHSLC tide gauges, even though in the course of this work a number of possible cases have been identified. (Some cases have also been occasionally reported in the literature: Valladeau et al. (2012) highlighted likely datum errors of order 10 cm at the Balboa, Panama, tide gauge between 2002 and 2006, which we can confirm.) As part of the JASL production of RQ data, any non-UHSLC problems are being referred to the data originators, which is a part of routine quality-control procedures to mitigate instrument and data-processing issues. Here we use Alt–TG differences to explore in detail some leveling problems that we identified at stations with the auxiliary metadata that is necessary for a successful investigation.

## Data and data-processing approach

Approaches to forming and studying Alt–TG sea-level differences^1^ have been widely discussed in the literature, e.g., see reviews by Wöppelmann and Marcos (2016), Kleinherenbrink et al. (2018), Oelsmann et al. (2021). Although there is obvious overlap in all approaches, many details differ and ours are briefly noted here. In our experience, we have found generally lower noise levels by using multi-mission gridded altimeter data and daily-mean tide-gauge data. Oelsmann et al. (2021) argue for the use of along-track altimetry. Other groups have their preferred methods (Wöppelmann and Marcos 2016). The advantages and disadvantages of each method need not be debated here.

### Altimeter data

Two sources of gridded altimetry, each produced by optimally interpolating satellite measurements in time and space, have been used in this work. We find that differences between the two can be useful for assessing reliability of proposed offsets and their associated uncertainties as well as the effect on resulting VLM determinations.

One altimeter source is the Data Unification and Altimeter Combination System (DUACS) delayed-time (DT-2021) product, an update to work described by Taburet et al. (2019); the data are distributed by the Copernicus Marine Service (CMEMS). These are gridded sea-surface height anomalies, produced daily on a 0.25$^{\circ }$ global grid and based on multiple satellite missions, generally from two to five simultaneously flying altimeters. The DUACS gridding algorithm used a temporal correlation scale ranging from 10 to 33 days, depending on latitude (Pujol et al. 2016). In light of that, we subsampled the data at 5 days.

Our second source of altimeter data, called NASAssh, is a product originally associated with the NASA project Making Earth System Data Records for Use in Research Environments (MEaSUREs). It consists of gridded sea-surface height anomalies with 5-day sampling on a (1/6)$^{\circ }$ grid. The grids were constructed with data from two altimeters at each time step—one from the TOPEX-Jason series and one from either ERS, Envisat, AltiKa, CryoSat-2, or Sentinel-3A. The TOPEX-Jason data were given greater weight in the gridding, which forced the mapped sea levels to conform more closely to the orbits and reference frames used for those missions. The TOPEX-Jason orbits were computed throughout the nearly three-decade time series in consistent fashion, with the same reference frame, gravitational models, geocenter models, and satellite tracking types (Lemoine et al. 2010; Zelensky et al. 2018). Further details are available in unpublished work by Zlotnicki et al. (2019) and, more briefly, by Ray et al. (2021, Appendix C).

Thus, while both altimeter products rely heavily on the TOPEX-Jason series, they differ in the inclusion of other altimeters. They also differ in their satellite ephemerides, tide models and some other geophysical corrections, and optimal interpolation algorithms. It is therefore a useful exercise to employ both.

At the time of writing, it is generally acknowledged that the early TOPEX data suffer from a small ($\pm 5$ mm) U-shaped drift (e.g., Valladeau et al. 2012; Watson et al. 2015; Beckley et al. 2017), although there is no consensus on the best way to resolve the problem (Legeais et al. 2018). The DUACS data release provides an optional TOPEX correction based on Alt–TG differences, which we apply (notwithstanding our philosophical desire to keep the two systems independent). The NASAssh data follow Beckley et al. (2017) in removing an internal altimeter calibration correction that they argue was flawed once the TOPEX altimeter’s point target response began drifting. For both altimeter products, global validation assessments are improved with these adjustments (e.g., Beckley et al. 2017).

**Table 1:** Estimated tide-gauge offsets in UHSLC Research Quality (RQ) data

UH ID	GLOSS ID	Station name	Required adjustments (mm)		
			Time	DUACS	NASA
5b	112	Majuro	2006.89	$56 \pm 7$	$50 \pm 7$
			2008.42	$-56 \pm 7$	$-50 \pm 7$
8b	119	Yap	2005.87	$-18 \pm 4$	$-19 \pm 5$
15b	140	Pape‘ete	2007.50	$18 \pm 4$	$5 \pm 7$
30a	–	Santa Cruz	1998.00	$-12 \pm 3$	$-11 \pm 6$
			2008.00	$32\pm 3$	$24 \pm 5$
104d	026	Diego Garcia	2006.50	$-41 \pm 9$	$-41 \pm 8$
105a	019	Rodrigues	2013.80	$-32 \pm 6$	$-32 \pm 6$
171a	046	Cocos$^\textrm{a}$	1994.00	$-104 \pm 8$	$-103 \pm 10$
			1995.00	$104 \pm 8$	$103 \pm 10$
			2000.45	$-26 \pm 3$	$-24 \pm 4$
211a	245	Ponta Delgada	2008.00	$-24 \pm 8$	$-35 \pm 9$
223e	253	Dakar	2005.00	$40 \pm 8$	$20 \pm 10$
803a	234	Rørvik	2007.00	$-97 \pm 11$	$-96 \pm 18$

Times are listed at a precision needed to reproduce our estimated offsets, but in general our Alt–TG technique cannot determine offset times to this stated precision

$^\textrm{a}$The 2000 adjustment at Cocos is not applied in Fig. 10b, because the offset may have arisen from tectonic processes

### Tide-gauge data

All tide-gauge data discussed in this paper are from the Joint Archive for Sea Level (JASL) RQ data at the UHSLC (Caldwell et al. 2015), as the archive existed at the end of January 2020, with the most recent data having been collected in December 2018. The RQ data have already undergone review for potential problems, including leveling errors that could affect elevation datums. Data from the UHSLC Fast Delivery (FD) database extend many of the RQ time series into subsequent years, but we do not consider the FD data here since those more recent data have not yet undergone as comprehensive quality control.

The tide-gauge data are in the form of daily means as produced at the UHSLC. Aliasing associated with sub-daily tidal signals has been almost completely eliminated by applying a special low-pass filter (Kilonsky and Caldwell 1991) to hourly data before daily means were computed.

To form Alt–TG differences it is critical that the tide-gauge and altimeter data be processed as consistently as possible. The gauge data have therefore been adjusted using the same ocean model of Carrère et al. (2016) that was applied to the altimeter data; the model aims to remove ocean variability from high-frequency (period $< 20$ d) wind and pressure loading and low-frequency inverted-barometer pressure loading. An additional benefit of this removal is that the ocean model is evaluated at the exact location of a tide gauge, so differences in sea level between the tide-gauge and altimeter locations are minimized, to the extent the model is capable of realistically simulating such differences; this reduces ocean “noise” in Alt–TG differences, allowing better delineation of any datum issues. The ocean model of Carrère et al. (2016) is a finite-element barotropic model released on a 0.25$^{\circ }$ grid. Future model development will have higher spatial resolution, so once that model is applied to altimetry the benefit of applying this correction to the tide-gauge data will grow.

**Table 2:** Vertical land motion estimates (mm y$^{-1}$), before and after offset adjustments

UH ID	Station name	DUACS altimetry		NASAssh altimetry		GNSS ID	Dist (km)	UNR$^\textrm{b}$	JPL$^\textrm{c}$
		Before	After	Before	After				
5	Majuro	$-0.33\pm 0.54$	$-0.42\pm 0.40$	$-0.44\pm 0.39$	$-0.51\pm 0.39$	MAJB	1.68	$-0.18\pm 0.72$	$0.53\pm 0.82$
8	Yap	$-0.61\pm 0.20$	$0.42\pm 0.31$	$-0.77\pm 0.20$	$0.25\pm 0.40$				
15	Pape‘ete	$-0.88\pm 0.23$	$-1.90\pm 0.37$	$-0.93\pm 0.27$	$-1.21\pm 0.59$	PAPE	0.66	$-1.57\pm 0.77$	$-1.61\pm 0.32$
30	Santa Cruz	$0.42\pm 0.27$	$-0.96\pm 0.25$	$0.13\pm 0.20$	$-0.83\pm 0.44$	GLPS	1.71	$-1.12\pm 0.42$	$-1.27\pm 0.14$
104	Diego Garcia	$-2.84\pm 0.71$	$-1.00\pm 0.67$	$-2.13\pm 0.65$	$-0.22\pm 0.65$	DGAR	3.40	$0.29\pm 0.55$	$-0.13\pm 0.29$
105	Rodrigues	$-3.37\pm 0.33$	$-2.35\pm 0.30$	$-2.78\pm 0.32$	$-1.76\pm 0.30$	RDRG	1.65	$-0.84\pm 0.83$	
171	Cocos$^\textrm{a}$	$-2.93\pm 0.53$	$-2.36\pm 0.22$	$-2.25\pm 0.54$	$-1.86\pm 0.27$	COCO	9.57	$-0.87\pm 0.60$	$-2.35\pm 0.48$
211	Ponta Delg	$-1.89\pm 0.41$	$-0.34\pm 0.78$	$-1.92\pm 0.57$	$0.36\pm 0.87$	PDEL	1.62	$-1.96\pm 0.51$	$-2.02\pm 0.36$
223	Dakar	$2.24\pm 0.31$	$-0.02\pm 0.61$	$0.97\pm 0.33$	$-0.16\pm 0.82$	FG02	0.34	$0.22\pm 1.01$	
						DAKR	6.12	$-1.10\pm 0.68$	$-0.49\pm 0.42$
803	Rørvik	$-1.56\pm 0.66$	$4.07\pm 0.92$	$-1.48\pm 0.95$	$4.03\pm 1.52$	VIKC	0.78	$3.62\pm 0.57$	

$^\textrm{a}$Cocos ‘after’ trends are for data since 2000

$^\textrm{b}$UNR: University of Nevada, Reno (Blewitt et al. 2016)

$^\textrm{c}$JPL: Jet Propulsion Laboratory (Heflin et al. 2020)

For the same reason, the tide-gauge data were also corrected for long-period ocean tides and the pole tide. The former is a mix of dynamic (but nearly equilibrium) waves and self-consistent equilibrium waves, spanning periods from 9 days to 18.6 years. The 18.6-y term is the most important; neglecting to account for that wave could potentially lead to Alt–TG differences with almost 2 mm y$^{-1}$ trends over 9 y at the equator (and twice that in polar regions). The pole tide has two dominant periods, at 12 and 14 months; the latter is more important in this context because annual cycles are generally removed subsequently anyway (see below). Our calculation of the pole tide follows Desai et al. (2015), but with a definition of the earth’s mean pole from Ries and Desai (2017). Note that only the ocean tides should be removed from the gauge data, whereas both ocean and solid tides were removed from the altimeter data.

Finally, in light of the inherent temporal filtering of the altimeter data during the gridding step, the tide-gauge data were also subjected to a series of low-pass filters. We did not attempt to follow the exact temporal decorrelation scales of the altimeter optimal interpolators for this, which are complex (Pujol et al. 2016), but instead used a series of low-pass filters with cutoff periods between 21 and 35 days and examined each during the subsequent differencing.

### Alt–TG difference time series

In terms of understanding tide-gauge errors, an emphasis on VLM, rather than its negative (TG–Alt), can be a (minor) source of confusion: a positive offset in a tide gauge’s reported sea level is associated with a negative offset in implied VLM. However, the advantages of consistent discussions in terms of VLM, including direct comparisons against independent VLM measurements, outweigh the disadvantages of possible sign confusion when assigning tide-gauge offset errors. (Thus, if any datum corrections are made to tide gauges based on the analyses here, then the sign of the adjustments should be opposite ours.)

For any given tide gauge, multiple time series of Alt–TG differences were computed using data from about three dozen grid points closest to the gauge. A similar approach was employed by Kleinherenbrink et al. (2018). At each grid point, up to 10 different low-pass filters were applied to each Alt–TG time series. The seasonal cycle (annual plus semiannual cycles) was removed from each difference time series, as it is not uncommon that the seasonal cycle varies rapidly from a coastal gauge to deeper water (Strub et al. 1987; Vinogradov and Ponte 2010), and this variation is not germane to the work here.

Of these several hundred Alt–TG time series for each tide gauge, we selected the one of smallest variance. This final Alt–TG time series was then also subjected to an additional 7-point median filter, for suppression of remaining outliers and general noise reduction, which is useful to subsequent analysis. With 5-d sampling, this median filter corresponds to 35 d full-width.

### GNSS data

The estimates of VLM are an important result arising from the Alt–TG differences. Whenever possible, these estimates are compared with independent VLM estimates from global navigation satellite system (GNSS) measurements, taken from either Blewitt et al. (2016) or Heflin et al. (2020); these two works used strikingly different approaches to derive VLM from GNSS data. Our quoted GNSS uncertainties are taken directly from those authors. In most cases, the uncertainties of Blewitt et al. are a good deal larger than those of Heflin et al. even for essentially the same time series.

The VLM rates from both GNSS groups are periodically updated as data are reprocessed and as time series lengthen. The rates given here were extracted from the University of Nevada website on December 14, 2022 and from the Jet Propulsion Laboratory website on February 22, 2022.

Aside from these linear rates, it can be useful to examine the GNSS time series of estimated daily positions, as these can reveal nonlinear VLM or other aspects of the data. Relevant GNSS time series, extracted from the solutions of Blewitt et al. (2018), are shown in the Supplemental Information.

Many of these GNSS time series are seen to be gappy or short and rarely span the full range of times of any tide gauge. We have therefore checked all suspected tide-gauge offsets against the global earthquake catalogs of the EarthScope Consortium,^2^ although other causes of rapid VLM are also possible.

## Identification and estimation of reference-level offsets

In any time series of Alt–TG differences, times and magnitudes of possible offsets—either temporary over short intervals or permanent—can in principle be detected by completely automated means. For example, Heflin et al. (2020) have developed an algorithm for this in their analysis of daily GNSS position data, and considerable work has been published on the GNSS problem (e.g., Vitti 2012; Gazeaux et al. 2013; Montillet et al. 2015). The problem is a common one with myriad applications (e.g., Tsay 1988; Reeves et al. 2007). In the context of Alt–TG data, see recent work by Oelsmann et al. (2022). Reliable solutions depend on careful statistical analysis of the underlying noise, especially if the offset times are unknown (Li and Lund 2015; Oelsmann et al. 2022). In this work, we instead rely on subjective identification of offsets and their associated times. Objective methods are then used to determine the magnitudes of offsets.

The precision possible for the identification of times is no better than a few weeks and possibly even as poor as two months. This is due in large part to the temporal decorrelation scales used in gridding of the altimeter data, but also to the noise levels in the data. Comparison with station maintenance records, and notably any recorded dates for sensor replacements, can provide additional evidence to narrow the temporal uncertainty, but here we used that information only afterward, as we attempted to understand identified offsets.

At any identified offset(s) in the Alt–TG time series, we have computed the offset amounts by least squares, simultaneously solving for a linear trend across a section that includes all offsets and extends (usually) to the beginning and end of the time series. An exception to this approach arises when temporary offsets of no more than (say) two years occur; in those cases we solved for a single offset over the short time period rather than two independent offsets. Again the choice of when this could be done was subjective.

Standard errors were computed for all estimated offsets and trends. We allowed for serial correlation, assuming an AR(1) process with an auto-correlation computed from the final residuals, allowing for gaps in the time series. An effective number of degrees of freedom and resulting inflation factor for uncertainty estimates follows from the lag-1 correlation coefficient (e.g., Lee and Lund 2004). Oelsmann et al. (2022) considered other possible noise models for Alt–TG data and concluded that AR(1) was acceptable.

Allowance for AR(1) serial correlation is still an internal assessment of uncertainties, and no accounting is made here of possible additional systematic errors, which could arise from either measurement system. One of the largest neglected error sources, in both altimeter and GNSS systems, arises from uncertainties or instabilities in the terrestrial reference systems, in both origin and scale (Wöppelmann and Marcos 2016). Because of this, Ballu et al. (2019), for example, inflate their uncertainties in estimated VLM by 0.5 mm/y (origin error) and 0.3 mm/y (scale error), a significant factor. Ablain et al. (2017) argue that local altimeter trend errors can be as large as 1–2 mm/y from orbit errors and 0.5$-$1.5 mm/y from tropospheric path delay errors. None of our VLM results below disagree with GNSS at such large levels, with the exception of anomalous results for one tide-gauge station at Ponta Delgado.

**Fig. 1:**
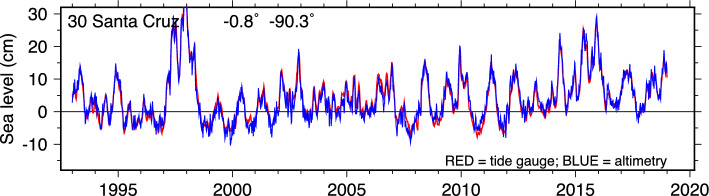
Sea level at Santa Cruz (Galapagos Islands) as observed by satellite altimetry and by the island tide gauge. The gauge data are from daily means, subsequently low-pass filtered to match the temporal scales of the gridded altimeter data. The high sea-level anomaly in 1997 is a manifestation of the strong 1997 El Niño on eastern tropical Pacific sea level

**Fig. 2:**
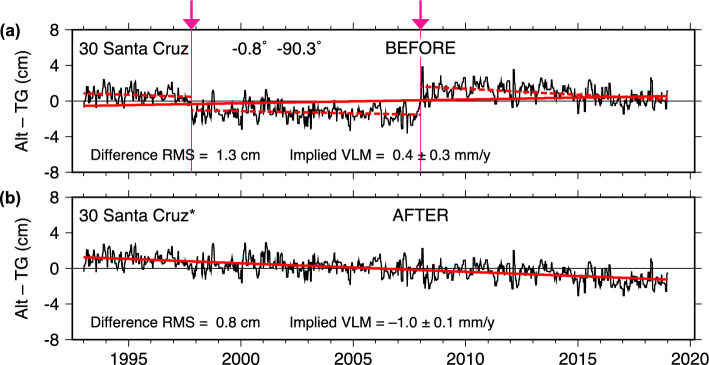
The Alt–TG time-series difference for the Santa Cruz (Galapagos Is.) tide gauge from DUACS altimetry, **a** before and **b** after adjustment of the tide-gauge data. Arrows at top mark times of the proposed adjustments and correspond with the times given in Table 1. Dashed red lines are least-squares fits to data falling between offsets. Solid red lines are fits to the whole time series; their slopes define the implied VLM. Uncertainty in VLM is based here on only the displayed data, whereas VLM uncertainties in Table 2 also account for associated uncertainties in estimated offsets

## Results

We present ten case studies in this section. All the estimated offsets are gathered together in Table 1, and the implied VLM from the Alt–TG data, before and after offset adjustments, are gathered in Table 2. Uncertainties listed in both tables correspond to one standard error. Table 2 also includes VLM trends from one or more nearby GNSS estimates, if available. Some older or shorter GNSS time series, not included in Table 2, are available for comparison in the Supplement.

The following figures are all based on DUACS altimeter data. Tables 1 and 2, however, include results from both DUACS and NASAssh data, since considerable interest lies in their mutual consistency.

### Santa Cruz

The case of Santa Cruz, in the Galapagos Islands, is probably one of the most convincing, even though the offsets are very small. It is thus a good case with which to begin.

The sea-level curves from altimetry and the filtered tide-gauge data are shown in Fig. 1. At the scale of the figure, the agreement is impressive. The RMS difference between the two curves is 1.3 cm. Small differences between the two curves are more readily seen in the Alt–TG series shown in Fig. 2a. In that figure, two probable offsets are apparent, one in late 1997 and one near the beginning of 2008.

The two offsets in Fig. 2a are very small: both altimeter datasets find only 1 cm for the first offset, and approximately 3 cm for the second. That such small offsets can be identified is due to the good agreement between altimetry and tide gauge at this location, resulting in the very low noise levels in the Alt–TG time series.

After adjusting the tide-gauge series by the estimated offsets, the Alt–TG differences (Fig. 2b) are remarkably clean (low noise) and fall nearly along a straight line. The RMS of the data dropped from 1.3 to 0.8 cm, the lowest of all cases here examined. Whereas a linear fit to the unadjusted DUACS series gave a small positive VLM trend ($0.42 \pm 0.27$ mm y$^{-1}$), the adjusted data indicate subsidence ($-0.96 \pm 0.25$ mm y$^{-1}$). The NASAssh data are consistent with these estimates within given error bars (Table 2).

The three individual segments of the original time series between the two jumps (dashed lines in panel a) have the following trends:

1993–1998: $$-0.84 \pm 0.98$$ mm/y 1998–2008: $$-0.50 \pm 0.38$$ 2008–2019: $$-1.33 \pm 0.37$$

with uncertainty understandably largest for the short initial section. All three indicate subsidence, and all are in agreement within (2$\sigma$) error limits with the trend of the full adjusted time series, but not with the original trend.

A small subsidence at Santa Cruz, as implied by Fig. 2b, is also consistent with GNSS station GLPS, with VLM estimates of $-1.12$ and $-1.27$ mm y$^{-1}$, both in excellent agreement with that inferred from the adjusted Alt–TG data. Moreover, the GNSS time series (Supplemental Figure S1) shows no indication of earthquakes or other anomalous VLM at the times of the two offsets.

The internal consistency between the three subsections and the final adjusted time series, all showing small subsidence, and the external consistency with the GNSS rates, build a convincing case that the proposed two offsets in the Santa Cruz tide gauge are legitimate, even though they are very small.

We suspect both offsets are related to instrument changes. The first and smaller offset coincides almost perfectly with a station maintenance visit on November 4, 1997, during which two of three encoder sensors were moved. The high sea levels associated with the then-ongoing El Niño (evident in Fig. 1) subsequently interfered with the maintenance because one of two mechanical water-level switches used to calibrate the tide-gauge datum stayed flooded throughout many successive tidal cycles. A small ($\sim$1 cm) error is thus not unreasonable.

The second and larger offset is more complicated but appears to fall near the time that UHSLC made at least two leveling changes. Following a pier reconstruction, the first change occurred on September 29, 2007. Then on February 18, 2008, the primary channel of the tide gauge was changed from a float to a radar sensor, the station was resurveyed, and a new primary benchmark installed. Subsequent post-processing in 2015 resulted in readjustment of these earlier level shifts, along with a 1-cm shift in 2014 and a 4-mm shift in 2015. At the precision of our Fig. 2a, the latter adjustments appear satisfactory, but possibly not those near late 2007 to early 2008. In any event, the evidence for instrument-related causes for both offsets at Santa Cruz seems strong.

**Fig. 3:**
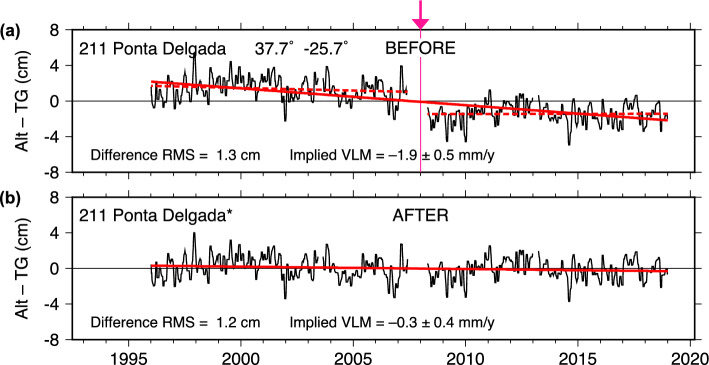
As in Fig. 2, for Ponta Delgada (Azores)

### Ponta Delgada

The case of Ponta Delgada (Azores) is similar to Santa Cruz, but less convincing because of inconsistencies in VLM. The original Alt–TG time series is shown in Fig. 3a, which indicates subsidence of about $-1.9$ mm/y, but the linear fit across each section before and after a short break in 2008 is unsatisfactory; it disagrees with the two dashed lines whose individual slopes are:

1996–2008: $$-0.60 \pm 1.12$$ mm/y 2008–2019: $$-0.04 \pm 1.10$$

both essentially zero. There is an apparent bias before and after the break in 2008, which we estimated (Table 1) as approximately 3 cm. With that offset, the trend in the full, adjusted time series is near zero ($-0.34 \pm 0.80$ mm/y) and thus in better agreement with the two segments before and after the 2008 break.

**Fig. 4:**
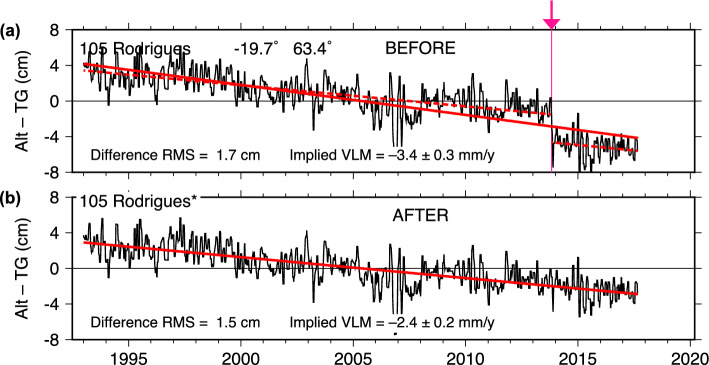
As in Fig. 2, for Rodrigues (Mauritius). In this case, the source of the 2013 jump has been traced to an instrumental problem: two calibration cables inadvertently switched during an October 2013 site visit

In this case, however, the GNSS data disagree. Station PDEL indicates subsidence at $-2.0$ mm/y (Table 2), which agrees closely with the original unadjusted Alt–TG series. The PDEL time series overlaps with the tide-gauge gap in 2008, but as Figure S2 (Supplement) shows, an unfortunate antenna change in early 2008 induced a large vertical offset, and thus the PDEL measurements cannot rule out a real, abrupt VLM offset around that time. Although no large earthquakes occurred at that time, a localized volcanic process cannot be ruled out (Okada et al. 2015), even though most of the volcanic unrest on São Miguel tends to occur on parts of the island away from Ponta Delgada (Mendes et al. 2017).

A DORIS receiver (station PDMB) is collocated with the PDEL station, and it yields a VLM estimate of $-1.52 \pm 0.20$ mm/y (F. G. Lemoine, personal commun., 2021), again suggesting subsidence, consistent with PDEL, although at a slightly slower rate. The DORIS time series is also gappy near 2008 and somewhat noisy (because early 2008 predates the Jason-2 satellite which markedly reduced DORIS noise). Therefore, neither GNSS nor DORIS is informative about small possible VLM jumps around 2008.

The GNSS and DORIS stations are located approximately 1.6 km from the Ponta Delgada tide gauge, on the roof of a relatively tall building. In such a geothermally active region, it is not inconceivable that VLM could differ rapidly over short distances. A geodetic station closer to the tide gauge would be valuable.

Station maintenance records do reveal important information about the break in 2008. The Ponta Delgada tide gauge was completely rebuilt during March 2008, when an Aquatrak acoustic gauge was replaced with a radar gauge with a pressure transducer backup. The sensor levels were then surveyed with respect to a tide staff, a technique somewhat prone to inaccuracy and one that UHSLC nowadays avoids using if possible. In light of that, it seems possible that a level shift did occur in 2008, even though a $\sim$3 cm shift is rather large. However, the inconsistency between the Alt–TG and geodetic VLM rates, from GNSS and DORIS instruments 1.6 km away, remains an important point and suggests caution before accepting the reality of a 3-cm reference-level shift during 2008.

**Fig. 5:**
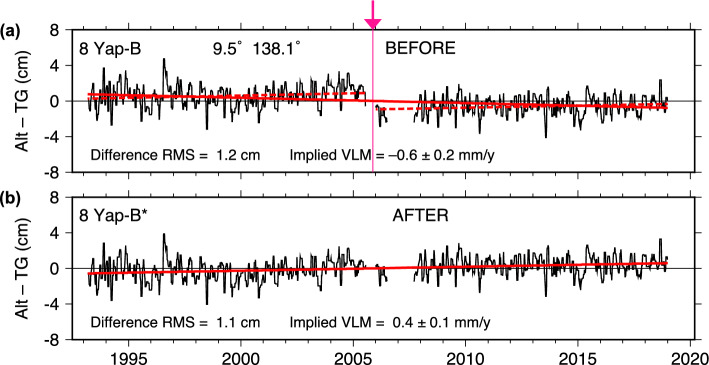
As in Fig. 2, for Yap (Micronesia)

### Rodrigues

The case of Rodrigues (Mauritius) appears straightforward, at least in retrospect. Figure 4a suggests a jump during the latter part of 2013. Both altimeter datasets give identical estimates for the offset: $-32 \pm 6$ mm (Table 1). The adjusted Alt–TG series (Fig. 4b) reduces the trend, but it still indicates significant subsidence: $-2.35 \pm 0.30$ mm y$^{-1}$ (DUACS) or $-1.79 \pm 0.30$ mm y$^{-1}$ (NASAssh), consistent within error limits. These subsidence estimates are larger than that reported at GNSS station RDRG by UNR, but the uncertainty on that rate is large ($-0.84 \pm 0.83$ mm y$^{-1}$). In fact, the RDRG time series (Figure S4) is short, gappy, and somewhat erratic; it is essentially inadequate for present purposes.

The Rodrigues case is one in which we have successfully tracked down the source of the offset problem. We discovered that, during the course of a site maintenance visit in October 2013, electronic cables to two calibration sensors for the radar gauge were inadvertently switched. This caused sea levels to be reported too high by the separation distance between the two sensors, which is 43 mm. The Alt–TG analysis found a slightly smaller estimate for this offset ($32 \pm 6$ mm). But the $\pm 6$ mm represents a 1$\sigma$ standard error, so within a 95% confidence interval the altimeter analysis rightly recovered the offset error. The Rodrigues tide gauge data in the UHSLC RQ archives will soon be corrected for this error, with metadata updated accordingly. (Data in the FD archive have already been corrected.)

### Yap

It is not uncommon for reference levels to shift by small amounts when a tide-gauge time series is interrupted, as in the previous example at Ponta Delgada. Data gaps normally occur because of equipment malfunctions or equipment replacements, which, as noted above, are when leveling problems are most likely to arise. Several more examples of likely offsets following data interruptions can be highlighted.

**Fig. 6:**
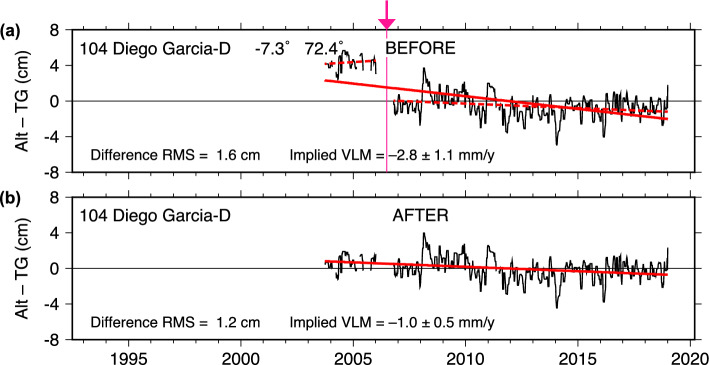
As in Fig. 2, for Diego Garcia

The Alt–TG series at Yap (in the Federated States of Micronesia) is shown in Fig. 5a, where a small offset is suggested after the short break in late 2005. The offset is slightly less than 2 cm—very small, but with excellent agreement between the two altimeter datasets (Table 1). The offset is close to the times of two separate site visits to Yap, after which sensor levels were adjusted by tide-staff readings, with a second adjustment of 8 cm noted in the records for reasons not readily transparent to us. There is also a discrepancy in the station records, with one record stating that in 2005 the primary data channel for the gauge was changed from a float system to a radar, but a second record stating that the sensor change occurred in 2007.

Aside from reference-level uncertainties related to either staff readings and/or sensor replacements, the main evidence that the Yap offset is real relies on the implied VLM slope and its consistency with the slopes before and after the break. A trend fit to the unadjusted DUACS Alt–TG series indicates subsidence: $-0.61 \pm 0.20$ mm y$^{-1}$, whereas trends before and after 2006 are both near zero but slightly positive:

1993–2005.87:   $$0.40 \pm 0.46$$ mm/y 2005.87–2019:   $$0.44 \pm 0.40$$.

The trend in the full, adjusted time series matches the two segments: $0.42 \pm 0.31$ mm y$^{-1}$. Although small and barely above noise levels, the adjustment in 2005 does improve the overall (internal) VLM consistency of the Alt–TG time series.

To our knowledge, there are no geodetic measurements of VLM in the area to which comparison can be made. This is doubly unfortunate since Yap is located in a region of the Pacific prone to large earthquakes. The question naturally arises whether the 2005 offset is from loss of reference level or from an earthquake. According to the EarthScope catalog, a Mw 5.7 quake did occur about 100 km from the tide gauge on September 2, 2005. On the other hand, a larger Mw 5.9 quake located only 30 km away occurred October 6, 2011, and Fig. 5 shows no indication of any offset associated with that quake. This suggests that the similar timing of the tide-gauge offset and the smaller quake in late 2005 may be mere coincidence. But we cannot definitively rule out a causal relationship as the amount of coseismic uplift depends not only on magnitude and distance but also on the nature and precise location of the earthquake.

**Fig. 7:**
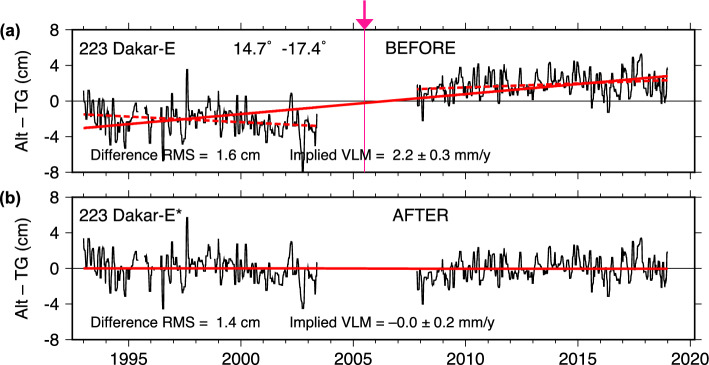
As in Fig. 2, for Dakar (Senegal)

**Fig. 8:**
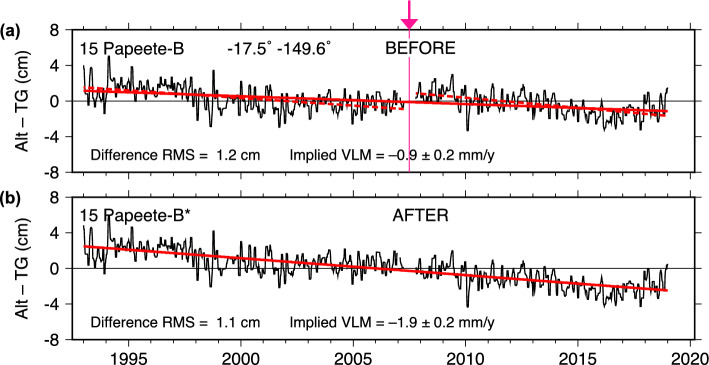
As in Fig. 2, for Pape‘ete (Tahiti, French Polynesia)

### Diego Garcia

The case of Diego Garcia, in the middle of the Indian Ocean, is similar to Yap, in that an apparent offset occurs in conjunction with a data gap. The data interruption in 2006 required a site maintenance visit to resume station operation. Figure 6a shows a clear jump at that time, much larger than the one at Yap, estimated at 41 mm (Table 1). Although the section before the jump is too short to give a reliable estimate of pre-2006 VLM, the fitted trend to the whole, unadjusted series, with slope $-2.8 \pm 1.0$ mm/y, yields a clearly unsatisfactory fit. The adjusted data in panel (b) still indicate subsidence at the gauge, but of much smaller magnitude.

The adjusted Alt–TG data from NASAssh yields a VLM near zero. The discrepancy between the DUACS and NASAssh final VLM trends, amounting to about 0.8 mm/y, is here the largest of our ten case studies, although the uncertainties (approximately $\pm 0.65$ mm/y for both) are large enough that both trends must be considered consistent. The nearby geodetic station DGAR gives VLM near zero (Table 2), in good agreement with the NASAssh value. The GNSS time series (Figure S5) also gives no indication of an offset around 2006, suggesting the offset is indeed of tide-gauge origin.

Station records for Diego Garcia indicate a large number of adjustments were also needed for the data between 2010 and 2018 as different sensors were intercompared and previous suspect calibrations were corrected. The adjustments were made during the 2019 update to the RQ data. So far as the satellite altimetry can assess them, these post-2010 adjustments were done accurately.

### Dakar

The case of Dakar (Senegal) is similar to Yap, but with a much longer data gap of about four years. After the gap, a radar gauge replaced the previous Aquatrak acoustic gauge. The original differences with DUACS altimetry (Fig. 7a) imply a large uplift: $2.24 \pm 0.31$ mm y$^{-1}$, but the two segments before and after the data gap dispute that ($-1.14 \pm 1.01$ and $0.87 \pm 0.70$), although with large uncertainties, especially in the Aquatrak section of the time series. Applying an estimated shift to the tide gauge of 4 cm after the break brings the final trend down to zero: $-0.02 \pm 0.61$ mm y$^{-1}$, about midway between the trends of the two segments. Ostanciaux et al. (2012) previously reported an uplift at Dakar of 1.835 mm y$^{-1}$ based on Alt–TG data, slightly less than our trend computed from the unadjusted data, but based on a shorter time series.

Unfortunately, the estimated jump from the NASAssh dataset is only half as large, at 2 cm. This is reflected in its original slope of 0.97 mm y$^{-1}$, which is only half the uplift derived from the DUACS data. The end results, however, are in agreement, with the NASAssh final trend also not statistically different from zero: $-0.16 \pm 0.82$ mm y$^{-1}$.

A vertical motion at Dakar near zero is in good agreement with the GNSS result obtained at station FG02 ($0.22 \pm 1.01$ mm y$^{-1}$) but with large uncertainty from an erratic time series (Figure S3). More precise estimates are available from station DAKR, but that station is twenty times farther from the tide gauge (300 m versus 6.1 km); the data at DAKR imply small subsidence (Table 2).

We conclude there is likely a reference shift in the tide gauge, probably between 2 and 4 cm, since all trends become more consistent with an adjustment. An ongoing uplift at Dakar of more than 2 mm/y, as implied by the original unadjusted DUACS data, does appear unlikely, as it would be in disagreement with both geodetic stations, as well as with the VLM before and after the four-year data gap.

**Fig. 9:**
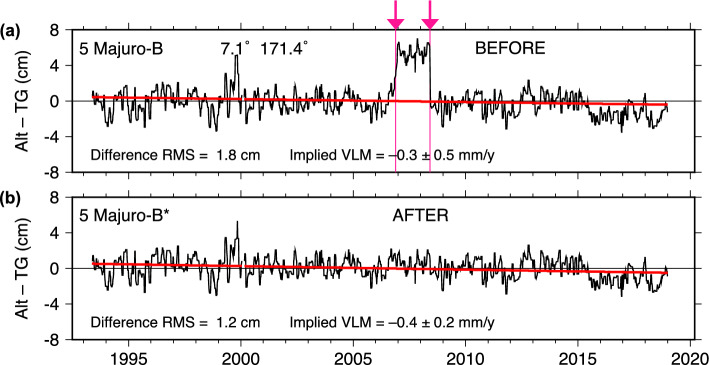
As in Fig. 2, for Majuro (Marshall Islands). Tide gauge corresponds to UHSLC RQ data as of 2022; updated data now available from the Australian Bureau of Meteorology no longer have the temporary offset seen in **a**

### Pape‘ete

The situation at Pape‘ete (French Polynesia) is similar to several of the other locations, with a possible offset in 2007 coinciding with a short break in the time series (Fig. 8). The break occurred when the station maintenance was transferred from NOAA to UHSLC, whereupon an Aquatrak acoustic gauge was replaced by a Vega radar gauge. The next year (2008 plus two months of 2009) the primary channel of the tide gauge was temporarily switched to a pressure transducer before being returned to the radar sensor.

A datum shift at the 2007 break, while small at 18 mm as estimated from DUACS data, turns an initial small subsidence of $-0.88$ mm y$^{-1}$ into a larger subsidence of $-1.90$ mm y$^{-1}$, which is in better agreement with the trends in the two segments either side of the break ($-1.70 \pm 0.48$ and $-2.25 \pm 0.60$ mm y$^{-1}$, respectively). The final VLM is also in better agreement with the GNSS estimates of $-1.6$ mm y$^{-1}$ at station PAPE. It is interesting to note that about ten years ago, all geodetic estimates of VLM near Pape‘ete were clustering around $-0.5$ mm y$^{-1}$ (Fadil et al. 2011), but with longer time series a larger subsidence appears to be indicated.

The datum offset in the NASAssh time series is smaller than DUACS, and in fact it is consistent with no offset at all. Yet accepting its value (5 mm) again moves the final VLM toward greater subsidence, from $-0.93$ to $-1.21$ mm y$^{-1}$. The final VLM rates from both adjusted Alt–TG series are thus in better agreement with the PAPE GNSS estimates.

Nonetheless, this small estimated offset is pushing the limits of the technique, even for a time series with relatively small noise levels, especially when the NASAssh data indicate that no offset is warranted. Suggestions of slight nonlinear curvatures in both sections before and after the break also lessen confidence in the reality of the small offset—unless, of course, that implied nonlinear VLM motion is real. In fact, there is some evidence for nonlinear motion in the GNSS time series (Figure S6): a large inflection at station TAH1 is clear around 2004, and a much smaller inflection at station PAPE may exist around 2016. Most of the Alt–TG curvature does not match, but the 2016 inflection does.

### Majuro

We now turn to several non-UHSLC tide gauges in which the Alt–TG methodology successfully reveals problems which, to some extent, may be dismissed as mere communication issues between data centers. The results nevertheless still illustrate the utility of the Alt–TG methodology.

The Majuro (Republic of the Marshall Islands) tide gauge is maintained by the Australian Bureau of Meteorology (BOM), who contributed the data to the UHSLC archive but who also now distribute the data publicly via their website. The sea-level differences for this gauge are shown in Fig. 9a. An offset of about 5 cm, extending over approximate times November 2006 through May 2008, is easily identified in the plotted differences. This feature is so unusual that it is almost certainly a temporary bias in the tide-gauge data. The least-squares estimate for the jump is $56 \pm 7$ mm. The corresponding jump for the NASAssh data is $50 \pm 7$ mm, consistent within error bars.

Because the jump is localized, it has ultimately little effect on the overall trend of the Alt–TG time series, changing the slope by only 0.1 mm/y. The variance of the time series, of course, is noticeably reduced (RMS dropping from 1.8 to 1.2 cm).

After this offset was discovered, further investigation revealed that the Majuro data now available from BOM no longer have this temporary (18-month) offset. It is unclear at this point when the data were corrected by BOM, but it must have occurred sometime after August 2013.^3^

At least for this simple case, the UHSLC RQ dataset can be readily updated, making an easy fix for a problem nonetheless originally (and then again recently) identified by the Alt–TG methodology. By comparing the old and new hourly tide-gauge series, we can also pinpoint exactly the affected times and bias. According to the BOM data, the bias is 53 mm, exactly midway between the DUACS and NASAssh estimates of 56 mm and 50 mm, respectively. The bias has a brief ramp-up of 6 h, with starting time November 20, 2006, and stopping time May 31, 2008, very close to our approximate estimated times given in Table 1.

### Cocos Island

**Fig. 10:**
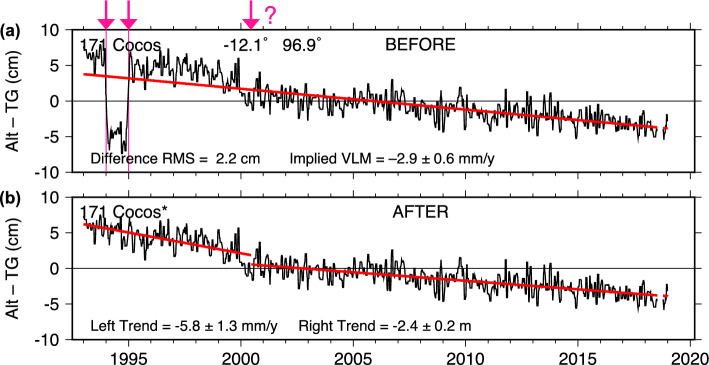
As Fig. 2, for Cocos (Keeling) Island. A large 10-cm bias in **a** is confined to calendar year 1994. A possible offset of 2.5 cm in early 2000 was left in the adjusted data, since it could conceivably be associated with a large 2000 earthquake near Cocos. Updated data now available from the Australian Bureau of Meteorology no longer have the 1994 offset seen in **a**

The Cocos (Keeling) Island tide gauge is also maintained by BOM. Similar to Majuro, a temporary offset was found in those tide-gauge data (Fig. 10a). In this case, the offset of about 104 mm was confined to the calendar year 1994. Present-day BOM data confirm the offsets seen in our Alt–TG analysis. Both Majuro and Cocos Island data will be updated in the UHSLC archives based on revised BOM data.

In addition to the 1994 problem, there is a suggestion in Fig. 10a of another possible offset around mid-2000. Both altimeter datasets yield an estimated offset of about 25 mm. However, we have left that offset in the adjusted data of panel (b), because of the strong possibility it is real VLM induced by tectonic influences near Cocos Island. On June 18, 2000, a large (Mw 7.9) earthquake occurred approximately 200 km from the tide gauge. GNSS solutions of Blewitt et al. (2018) show a clear offset in horizontal motion from the quake at station COCO, although the vertical is more confused, with little indication of co-seismic motion but possible post-seismic motion over the following years of at least 2 cm. Note too the significant differences between UNR and JPL trends estimated from the GNSS data: $-0.87$ versus $-2.35$ mm/y (Table 2). In light of the trend variability seen in the GNSS time series (see Figure S7), such large differences in mean trend are not too surprising. In comparison with other sites discussed here, the VLM at Cocos Island is complicated and nonlinear, somewhat similar to the complex tectonic deformations seen nearby in Indonesia (e.g., Fenoglio-Marc et al. 2012).

### Rørvik

**Fig. 11:**
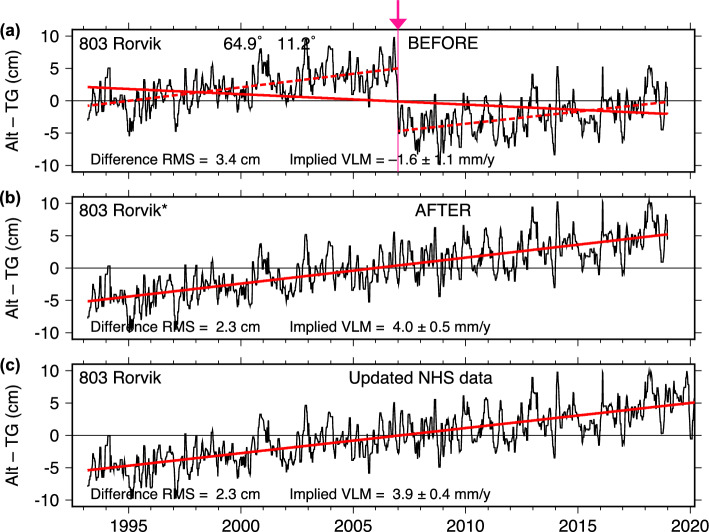
As in Fig. 2, for Rørvik, Norway. The tide gauge data in **a**, **b** correspond to UHSLC RQ data as of 2022; updated data now available from the Norwegian Hydrographic Service no longer have the offset in 2007

The tide-gauge data at Rørvik, Norway, shown in Fig. 11a, have a clear jump in Alt–TG differences at the beginning of 2007. Our two altimeter datasets give almost identical estimates for the jump: 97 and 96 mm. Similar to the cases of Majuro and Cocos Island, the data originators—in this case, the Norwegian Hydrographic Service (NHS)—have recently corrected errors and improved the quality of their data. The majority of the NHS corrections involved systematic errors related to their floating gauge system. The new data eliminate the 2007 jump.

Unlike Majuro and Cocos Island, however, the old and new NHS data differ by more than a constant offset. The older data also had a scaling error of approximately 3.6% in height, which is most easily detected by a running monthly tidal analysis, in which the amplitude of the semidiurnal $\hbox {M}_2$ constituent jumps from 78.8 to 81.6 cm (the phases are stable, indicating no timing errors are involved); in the newer NHS data the $\hbox {M}_2$ amplitude remains near 79 cm. Our adjusted data (Fig. 11b) did not account for this scaling, so we have run the Alt–TG analysis with the new NHS data (Fig. 11c), and we find the rescaled heights do not significantly affect results, at least at the scale of the plotting. Thus, our initial Alt–TG analysis was insensitive to the scaling problem and could not by itself have distinguished it from a simple bias, but it clearly detected the bias error (or the mean scaling error). Whatever the cause of the original post-2007 data problems, the updated NHS data should be considered definitive for this gauge and the UHSLC archive will be updated accordingly.

Although the gauge offset at Rørvik was large, nearly 10 cm, it is still encouraging that an offset of even that size can be detected there, because, at first glance, the location of the Rørvik tide gauge is not promising for the use of satellite altimetry. Rørvik is situated on a narrow strait, with large land masses on all sides. This likely explains the relatively large RMS difference of 2.3 cm (Fig. 11b), the largest of our ten examined cases.

## Discussion

The previous section describes evidence for which the Alt–TG differences uncover likely reference-level offsets in tide gauges, some of only a few cm. That an altimeter-based method can successfully detect errors of this magnitude is impressive, but it is important that readers are not misled: at many tide gauges the Alt–TG differences are too noisy to uncover offset errors of only a few cm. It is no coincidence that eight of our ten cases (excepting only Dakar and Rørvik) involve small islands in the open ocean, because the Alt–TG method typically works best at these locations. As stressed in the Introduction, the method relies on a common ocean signal successfully detected by both altimeter and tide gauge, and this is generally more difficult for tide gauges situated along the coasts of large land masses. There are three main reasons why errors are potentially exacerbated near land:

1. *Ocean dynamics.* Sea level at a coastal tide gauge can differ from that in the deeper open ocean for a variety of dynamical reasons (e.g., Woodworth et al. 2019). Many of the differences are relatively high frequency (e.g., storm surges), but low-frequency differences also arise, as predominant geostrophic dynamics in deep water will contrast with the ageostrophic dynamics near shore (Hughes et al. 2019). Many coastal gauges are also located near or in the mouths of major rivers, where variable discharge potentially generates buoyant flows trapped to the coast (Piecuch et al. 2018).

2. *Sampling.* Owing to the short spatio-temporal scales of many near-coastal and shelf phenomena, satellite altimetry, even with multiple satellites, may fail to capture the signals or may alias them in either time or space.

3. *Measurement error.* Errors in satellite altimetry are typically inflated near land owing to either instrumental problems (e.g., land contamination in altimeter or radiometer footprints) or inadequacies in required corrections; see discussions by Vignudelli et al. (2011) among others. The altimeter sampling can compound these errors by aliasing high-frequency errors to longer periods. For example, an inaccurate tide correction for an altimeter not only inflates noise levels in Alt–TG differences, but the (nominally diurnal or semidiurnal) error is then aliased to periods of many days. Aliased tide errors have been detected even in gridded products that use multiple satellites with differing repeat periods (Zaron and Ray 2018).

Most of these problems tend to be mitigated near small isolated islands, and the noise levels in Alt–TG differences are correspondingly reduced. Nonetheless, depending on tide-gauge location, problems can still arise (e.g., Williams and Hughes 2013). The tiny atoll of Minamitorishima is an interesting case in which Alt–TG differences are unusually noisy because of large (meter-level) wave setup at the tide gauge, which had been located directly behind a coral reef with large breaking swell (Ray et al. 2023); the gauge has been subsequently relocated.

These are all general characteristics, however, and in fact there are many non-island gauges with noise levels low enough to be amenable to this kind of analysis. Rørvik, on the coast of Norway, has the highest noise level of our ten cases, yet not so high that it can hide a nearly 10-cm jump. The main point is that the application of the Alt–TG analysis may fail for many tide gauges, especially continental tide gauges, if the suspected datum errors being sought are small.

When noise levels are sufficiently low and a likely offset in Alt–TG differences has been detected, the important question is whether the offset arises from an inadvertent loss of reference level at the tide gauge or it arises from real VLM such as an earthquake. If the latter, it represents a true jump in relative sea level, and in no circumstance should any “adjustment” of the tide gauge data be proposed. Independent measurements of VLM, such as from nearby GNSS receivers, are exceedingly helpful when addressing the question. Even if an event itself is not captured by a GNSS receiver, so that real VLM (e.g., an earthquake) can be ruled out, the consistency between rates of VLM from direct geodetic measurements and those implied from Alt–TG estimates, before and after potential tide-gauge adjustments, are important indicators of reliability. If internal and external VLM estimates become more consistent, the likelihood of an offset being real increases. By this measure, the evidence for a level shift at Ponta Delgado was weakened.

Even more critical are station maintenance records. When station records reveal offsets coinciding with site visits during which a sensor was modified or replaced, a small offset in reference level is certainly possible; and if the survey techniques that were employed to re-establish reference are now known from experience to be less than ideal (e.g., by reliance on a tide-staff reading), the likelihood of an offset increases further. Several of our ten cases fell into this category. A number did coincide with sensor replacements, and some (e.g., Yap in 2005 and Ponta Delgada in 2008) relied on staff readings. The best outcome, of course, is when Alt–TG analyses lead to discovery of instrumental or data processing errors that can be corrected at their source, as was the case with the Rodrigues tide gauge.

When no explanation for an Alt–TG offset can be discovered from station records, even if a proposed level adjustment leads to more consistent VLM estimates, then adjustment of the data by a primary archive center like JASL is difficult to justify. Our recommendation for these cases is that any tide-gauge adjustments, such as most of those given here in Table 1, should remain outside the archiving centers. This is consistent with our philosophy of maintaining independence between altimetry and tide gauges. Nevertheless, we can envision applications in which adjusted tide-gauge data can be employed, so long as potential users understand the limitations and associated risks inherent in the adjustments. How best to distribute those modified data, and in what form (one option is simply as adjustment tables as in Table 1), are questions for the sea-level community as a whole to resolve.

For our four resolved cases (Rodrigues, Majuro, Cocos, and Rørvik), we can report that the data archived at the Permanent Service for Mean Sea Level (Holgate et al. 2013) have already been corrected. The data in the GESLA-3 archive (Haigh et al. 2022) will be updated with its next release.

## Summary

The UHSLC archives two widely used databases of hourly and daily tide-gauge measurements: a Fast Delivery (FD) version for time-sensitive applications and a Research Quality (RQ) version for applications (e.g., sea-level rise) requiring greater certainty in the long-term stability of the vertical reference. Most records in the JASL RQ data do not contain detectable losses of vertical reference, but such errors do exist in the database, and they can be difficult to resolve, in part because the data are contributed by dozens of international agencies with varied objectives for collecting sea-level data. We have used the Alt–TG methodology to highlight ten cases of RQ time series in which the tide-gauge reference level has likely experienced an abrupt offset. Seven of these cases involve tide gauges operated and maintained by the UHSLC, which allows for cross-checking of potential non-physical offsets with station maintenance records and data-processing logs. We demonstrated how—in at least one case—this procedure can be used to identify and repair losses of vertical reference.

For that particular tide gauge, at Rodrigues, Mauritius, the Alt–TG method uncovered a 4-cm jump, which was eventually found to be caused by two calibration sensors whose cables had been inadvertently switched during a site visit. For three other cases (Majuro, Cocos, and Rørvik), offsets were uncovered which we subsequently discovered had already been corrected by the data originators even though the updated data had not yet entered the RQ archive. In most of the other cases, small offsets of only a few cm were found to occur in conjunction with a break in the tide-gauge time series, often associated with a change in sensor. Most of these are probably legitimate offsets unassociated with any geophysical process, although the case of Pape‘ete is not especially convincing because the offset is so small and two altimeter datasets disagree on its magnitude. The case of Ponta Delgado is also questionable because the implied VLM disagrees with nearby GNSS data; placement of a GNSS station properly collated at the tide gauge would be valuable for resolving that issue. On the other hand, the case of Santa Cruz is especially convincing even though the offsets are very small (only 1 and 3 cm), because site visits did occur around the times of offsets and because the implied VLM is more internally consistent and also externally consistent with nearby GNSS data.

Most of the discovered offsets are small, some only a few cm; the largest is 10.4 cm. The ability of the Alt–TG method to uncover such small errors is a function of the noise level in the differenced time series, and this depends critically on whether the altimeter and tide gauge are successfully measuring identical ocean signals. In general, this is more likely for tide gauges located on small islands in the open ocean and less likely for gauges surrounded by large expanses of land. These are only general statements, however, and we have found the method worked at the Rørvik tide gauge, which is situated in a narrow strait otherwise surrounded by land. While it does fail in some coastal environments (in the sense that Alt–TG noise levels are too large to detect small offsets), the use of satellite altimetry appears to be a powerful technique for quality control of modern (post-1992) tide-gauge data. As the IOC tide-gauge manuals recommend (UNESCO/IOC 2020), the UHSLC data are routinely checked for offsets using the Alt–TG approach, and the method is recommended as standard procedure for all tide-gauge operators and sea-level data centers.

### Supplementary Information

Below is the link to the electronic supplementary material.

Supplementary file 1 (pdf 9061 KB)

## Data Availability

DUACS altimeter data are available from the Copernicus Marine Service https://marine.copernicus.eu. NASAssh altimeter data are available from the Jet Propulsion Laboratory data archive https://podaac.jpl.nasa.gov/measures-ssh. Tide-gauge data are available from the University of Hawai‘i Sea Level Center https://uhslc.soest.hawaii.edu. The Australian tide-gauge data were originally distributed by the Australian Bureau of Meteorology; they are also available from http://www.bom.gov.au/oceanography/projects/abslmp/data. Earthquake information is available from the EarthScope Consortium at http://service.iris.edu/fdsnws/event/docs/1/. Additional datasets generated during this work are available from the corresponding author upon reasonable request.

## Footnotes

1 At least one publicly available system for tide gauge and altimeter comparisons is available through the University of Colorado: https://ccar.colorado.edu/altimetry.

2 http://service.iris.edu/fdsnws/event/1/. We examined only quakes of magnitude 5.0 and larger.

3 An e-mail dated 9 August 2013 from Pat Caldwell (UHSLC) to BOM first called attention to the 2007 Majuro problem as well as the 1994 Cocos problem.
