# Supplementary material for: Offsets in tide-gauge reference levels detected by satellite altimetry: ten case studies
Source: J Geod. 2023 Dec 3;97(12):110. doi: 10.1007/s00190-023-01800-7 (PMC10694126; doi:10.1007/s00190-023-01800-7)
Supplement: Supplementary file 1 — (pdf 9061 KB) [file 190_2023_1800_MOESM1_ESM.pdf]

Supporting Information for

" Possible offsets in tide-gauge reference levels detected by satellite altimetry: Ten case studies"

R. D. Ray[1], M. J. Widlansky[2], A. S. Genz[3], P. R. Thompson[2]

[1]NASA Goddard Space Flight Center, Greenbelt, Maryland, USA

[2]University of Hawai'i at Mānoa, Honolulu, Hawaii, USA

[3]NOAA, Honolulu, Hawaii, USA

Introduction

This supplement comprises a series of figures showing time series of GPS daily solutions for stations relevant to tide gauges discussed in the main paper. All GPS solutions here are from Blewitt et al. (2018) (the solutions are currently GPS only, as no other GNSS data are used). The digital data are available from the website of the University of Nevada at Reno at: `http://geodesy.unr.edu`. The daily solutions at UNR are continually updated; data shown here correspond to solutions as of 16 January 2023. Solutions are given in the IGS14 reference frame.

In addition to daily GPS solutions, each figure has one or more red vertical arrows marking time(s) of our proposed offset(s) in the associated relevant tide gauge. The arrows are consistent with the times shown in Table 1 of the main paper. In addition, each figure may also display vertical cyan lines which mark times of GPS equipment changes (e.g., replacement of receivers); many of these coincide with noticeable offsets in the GPS time series. Finally, each figure may display grey dashed lines which mark times of earthquakes that are potentially large enough and close enough to affect horizontal or vertical positioning. Earthquakes are marked if they satisfy an empirical criterion which was developed by Bill Hammond (personal communication, Jan. 2023) and is used on the UNR website. According to this criterion, an earthquake is included if the distance between its epicenter and the GPS station is less than $10^{(M/2-0.79)}$ km, where M is earthquake magnitude.

The figures—one for each tide gauge—may display GPS solutions for more than one station if several relevant stations are (or were) near the tide gauge. If that occurs, each station is displayed in different color and may (or may not) be offset by arbitrary amounts for display purposes. Table 2 includes linear VLM trends from the most relevant GPS station at each tide gauge. For the UNR data, these trends are based on the method described by Blewitt et al. (2016).

Contents

1. Figures S1 to S7, displaying relevant GPS solutions from Blewitt et al. (2018).

Corresponding author: R. D. Ray, `richard.ray@nasa.gov`

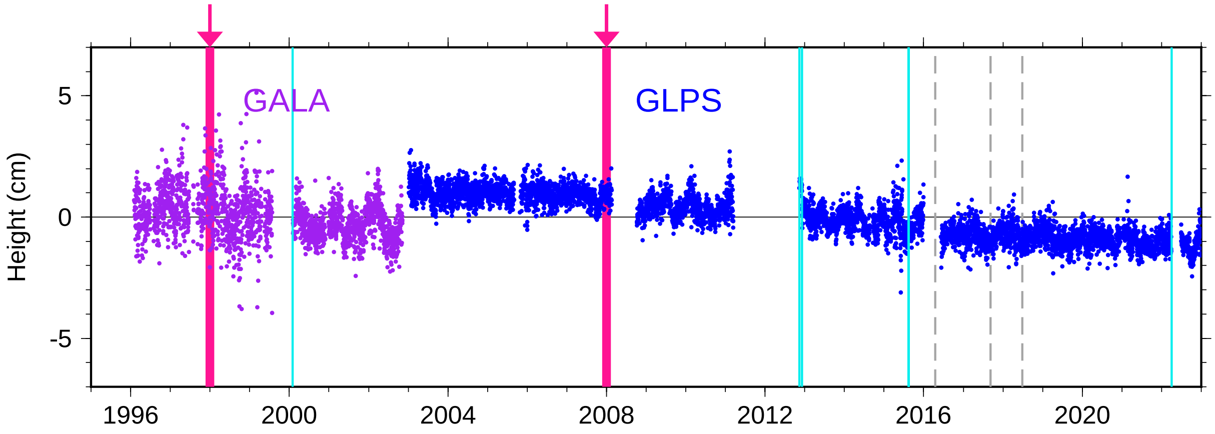

Figure S1. Daily GPS solutions for geodetic stations GLPS and GALA, near the Santa Cruz tide gauge. Station GLPS is 1.71 km from the tide gauge. Station GALA was 1.74 km from the gauge.

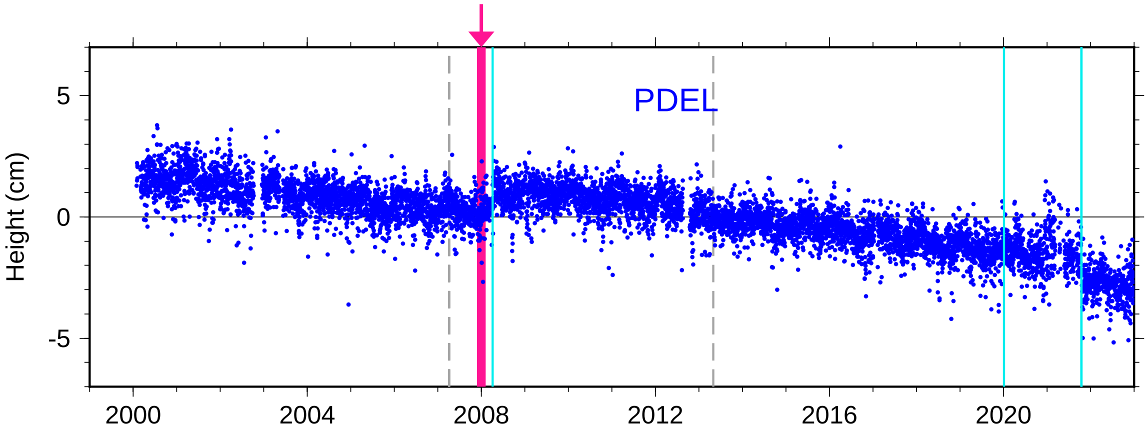

Figure S2. Daily GPS solutions for geodetic station PDEL, located 1.62 km from the Ponta Delgada tide gauge. An equipment change (cyan line) was unfortunately close to the time of the proposed tide gauge reference level offset.

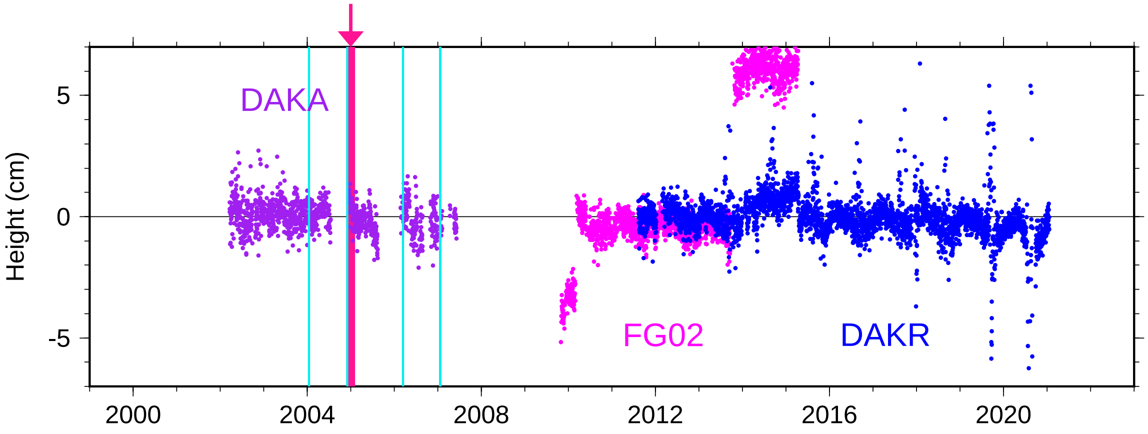

Figure S3. Daily GPS solutions for geodetic stations DAKR, DAKA, and FG02, near the Dakar tide gauge. Distances to tide gauge are: 1.2 km (FG02), 4.9 km (DAKA), and 5.4 km (DAKR).

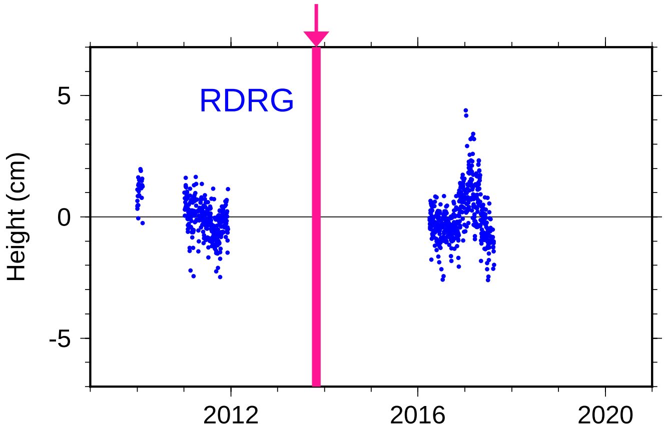

Figure S4. Daily GPS solutions for geodetic station RDRG, located 1.65 km from the Rodrigues tide gauge.

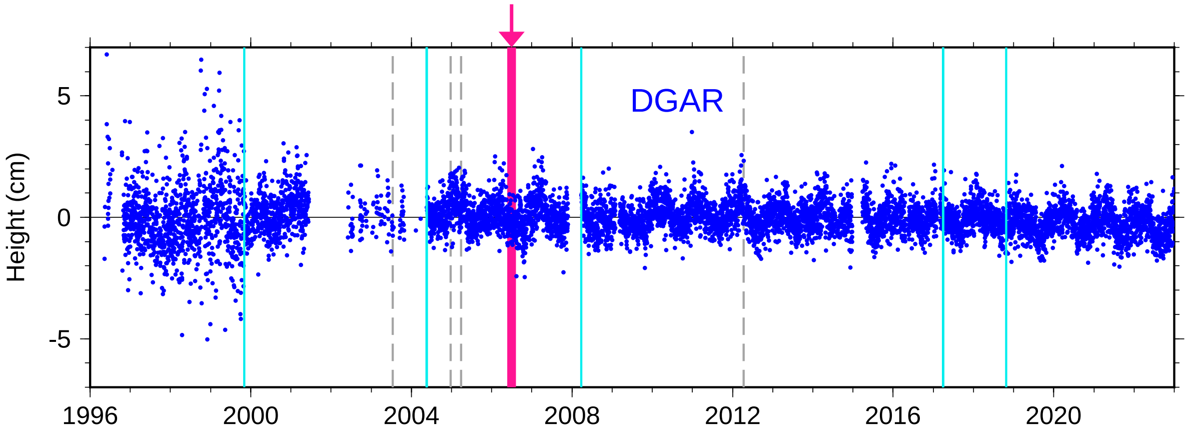

Figure S5. Daily GPS solutions for geodetic station DGAR, located 3.4 km from the Diego Garcia tide gauge.

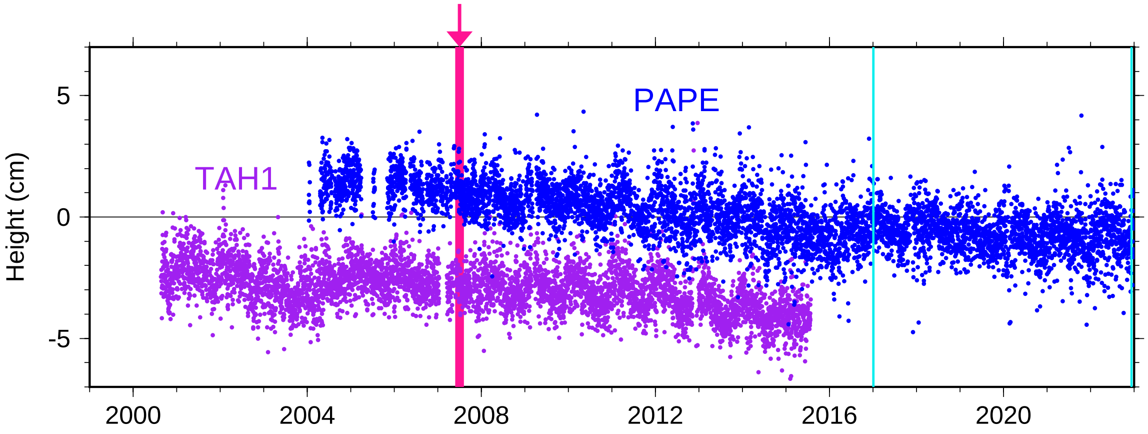

Figure S6. Daily GPS solutions for geodetic stations TAH1 and PAPE, near the Pape‘ete tide gauge. Station PAPE is 1.10 km from the tide gauge. Station TAH1 was 7.12 km from the gauge. For display purposes, the two time series are shown offset by arbitrary amounts.

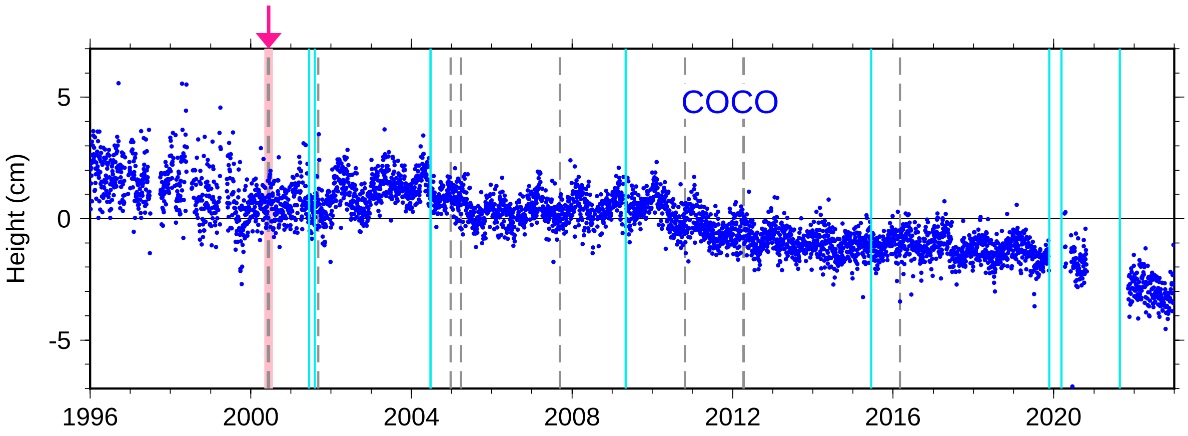

Figure S7. Daily GPS solutions for geodetic station COCO, located 9.6 km from the Cocos Island tide gauge. A suggested offset in the tide gauge data, at time marked by the pink line, coincides with a Mw 7.9 earthquake with epicenter 190 km from the COCO station. The earthquake caused a clear offset in the horizontal (not shown), but the vertical is not obvious. Overall the motion at COCO appears to be nonlinear, even after taking account of the many equipment changes (cyan lines).
